# Epidemiology of Acne Vulgaris and Its Association With Lifestyle Among Adolescents and Young Adults in Hail, Kingdom of Saudi Arabia: A Community-Based Study

**DOI:** 10.7759/cureus.9277

**Published:** 2020-07-19

**Authors:** Fawwaz F Alshammrie, Rasha Alshammari, Renad M Alharbi, Farida Habib Khan, Saud K Alshammari

**Affiliations:** 1 Medicine, University of Hail, Hail, SAU; 2 Medicine and Surgery, College of Medicine, University of Hail, Hail, SAU; 3 Community Medicine, College of Medicine, University of Hail, Hail, SAU

**Keywords:** acne vulgaris, epidemiology, lifestyle

## Abstract

Acne vulgaris is the commonest dermatological problem internationally and nationally. Its incidence is increasing every year in the Kingdom of Saudi Arabia (KSA). Though it is not a major health issue but it significantly affects the patient cosmetically, psychologically and socially. Previous studies have shown its association with lifestyle (age, diet, stress, sleep, smoking, exercise, obesity, etc.) and family history. By simple cost-effective lifestyle modification, its occurrence and late consequences could be minimized. Few hospital-based studies are done on this issue in Hail City, KSA. Hence present cross-sectional study was designed where data was collected by Google-Form from 484 residents of Hail City. Results have revealed that 65% of our respondents were suffering from mild to moderate Acne. Thirty percent of the respondents had BMI equal to more than 30. Majority of respondents (81%) had acne on whole face. Similarly, dairy products were also consumed by more than 50%. Majority of respondents (more than 50%) took chocolates, fast foods, oily foods and sea-foods. Nuts were taken quite often by 37% of respondents. Age between 21-25 years and oily skin have a highly significant association (p = 0.000) with development of acne. Other variables that show significant association with acne were being obese, stressful, irregular menstrual cycles and excessive intake of nuts.

Hence there is a need to address this issue in order to design recommendations for the general public to minimize the incidence and consequences of acne vulgaris by simple lifestyle modifications.

## Introduction

Acne vulgaris is the eighth most prevalent disease globally. Previous studies have found the prevalence of acne vulgaris 9.4% worldwide [1, 2]. The manifestations of acne may range from very mild lesions, called physiologic acne, to very severe findings in the form of abscess formation [3]. A study done in Hail, Kingdom of Saudi Arabia (KSA) in the period 2008-2014 for five years found that 20% of patients attending a dermatology clinic complain of acne vulgaris as compared to 12.43% acne cases in the same hospital, King Khalid Hospital, in the period from 1995 to 1997 [4,5]. This reflects that the incidence of acne vulgaris is increasing in the Kingdom. Another study conducted in a nearby region of Northern Saudi Arabia, Arar, revealed 54% prevalence of acne among adolescents [6,7].

Previous studies investigating the potential link between lifestyle and acne vulgaris have shown controversial results [8]. Moreover, literature review suggested that its prevalence varies with several genetic and environmental factors. A study done by Ballanger et al. found a strong association of causation of acne vulgaris to both genetic and environmental elements [9,10]. Positive family history of acne vulgaris is associated with severe form of acne manifestations, in addition to early onset of pre-pubertal acne [9]. Prevalence of moderate to severe acne among those having positive family history was 20% as compared to 10% in those lacking a family history of acne [10-12]. Further it is found by studies that severity of acne is strongly associated with positive family history [13].

Since early 1900s, the association between acne and dietary habits has been questioned in the literature [14]. Literature showed enough epidemiological link to consider the effect of diet on acne [6,7]. Hence, studies have suggested dermatologists to encourage patients with acne to avoid refined carbohydrates and shift to low glycemic index foods [8]. Recent change in lifestyle globally and its effect on diet has ultimately affected body mass index that is linked to increase the prevalence and severity of acne [10-12].

Although it is not a major health issue, but it can significantly affect patient cosmetically, psychosocially and socially. Moreover, causing a substantial impact on the quality of life. Limited studies have been done on acne in Hail region with no previous community-based studies investigating the epidemiology and the disease profile thoroughly, necessitating the need for this issue to be addressed in order to achieve a better understanding of this disease, thereby leading to a positive patient outcome.

Further as the previous studies report increasing prevalence of acne vulgaris during the past two decades in KSA; present study was done to assess the association of acne vulgaris with epidemiological factors (such as age, diet, stress, sleep, smoking, exercise, obesity, etc.) by conducting a community-based survey, as the previous studies done in Hail, were all hospital based.

## Materials and methods

Objectives

The objectives of this study are: to study the epidemiology of acne vulgaris and its association with lifestyle among adolescents and young adults in Hail, Kingdom of Saudi Arabia; to estimate the prevalence of acne vulgaris among adolescents and young adults in Hail; to describe the pattern of acne vulgaris among adolescents and young adults in Hail; to investigate the association of acne vulgaris with lifestyle, including diet, exercise, sleep, skin care products, and related risk factors; to list the risk factors predisposing to acne vulgaris among adolescents and young adults in Hail; to describe different management strategies for acne vulgaris used by adolescents and young adults in Hail.

Subjects and methods

Hail City is located in northwest of KSA; the city has a population of 400,000. This study was planned in the mid of year 2019. In September 2019, proposal was approved by IRB (Institutional Review Board) of University of Hail. A descriptive cross-sectional community-based study was done in Hail City between 15th of December 2019 and 15th of January 2020. A structured questionnaire was designed and translated in Arabic language. Researchers used RaoSoft, an online calculator, to estimate sample size. Minimum effective sample size came out to be 384, calculated at the confidence interval of 95% (RaoSoft, 2013). However, researchers were successful in achieving sample size of 484.

It was pretested on 10 randomly selected people. Purpose of this pilot testing was to assess the clarity of question and identify any ambiguous or lengthy questions, in addition to testing its applicability. It was then fed on Google Form. Link was sent to 500 residents of Hail City through a variety of websites and apps. But there were 484 responses. The objectives of the study were explained on the first page of Google Form. Their participation reflected their consent for the study.

The questionnaire consisted of 32 close-ended questions. The questionnaire included six important domains: socio-demographic data, presence of acne and pattern, lifestyle and coexisting pathologic factors or diseases, specific risk factors, and management strategies, that were used including both medical and home remedies. Questions about socio-demographic data included age, sex, weight, height, nationality, residence, and level of education. The prevalence and pattern of acne was investigated using subjective questions about presence of acne, family history, severity, pattern, coexisting chronic disease, and management. Moreover, there were questions targeting specific lifestyle behaviors such as smoking, sleeping hours, and physical activity. In addition, there were questions about events aggravating acne, skin care products and skin hygiene, and diet.

Data was entered and analyzed by Statistical Package for Social Sciences (SPSS) version 23 (IBM Corp., Armonk, NY). Descriptive analysis was done showing frequency and percentages. For analytical analysis, chi-square test was applied keeping the level of significance (p-value) <0.05.

## Results

**Figure 1:**
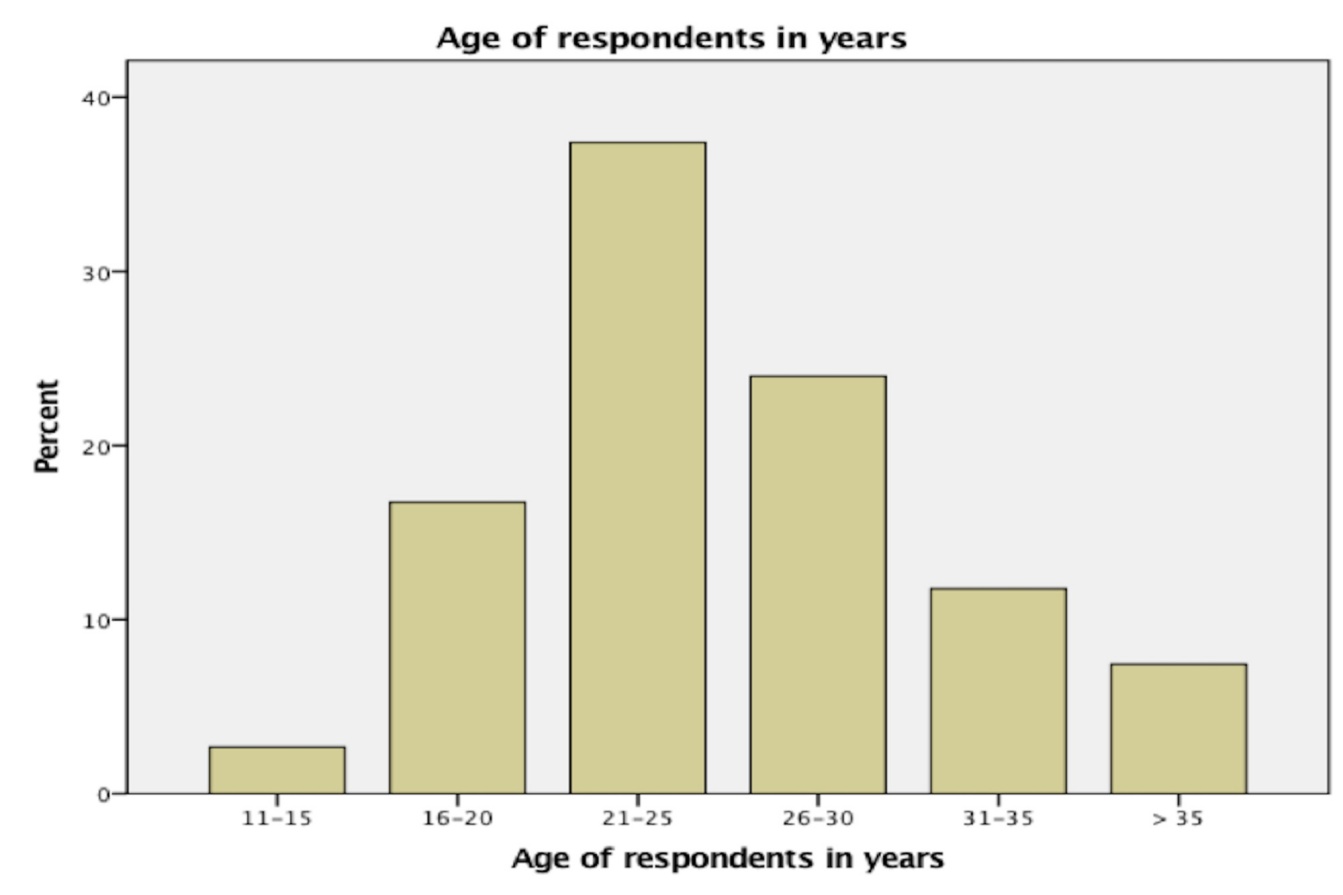
Age of respondents in years

Figure 1 shows age of respondents in range of five years interval. Most of the respondents (181/484 = 37.4%) were of the age group between 21-25 years.

Other demographic variables of respondents are shown in Table 1. Most of the respondents (84%) were females. Thirty-eight percent had body mass index (BMI) equal to more than 30. Most of the respondents were Saudis (98%) and residents of Hail City (93%). Seventy-six percent were qualified Bachelors. Table 2 shows that most of the respondents (53%) had combination of oily and dried skin, facial cleanser was used 3-4 times/week by 30% of respondents while majority (71%) did not use any sun-block cream. Ninety-one percent did not have any chronic disease (91%) and were non-smokers (94%). No sort of any physical exercise is being practiced by 56%, though almost the same percentage (50%) had enough sleep span of 7-9 hours/day. Among the female respondents, majority (68%) had irregular menstrual cycles.

**Table 1:** Demographic profile of respondents (n = 484)

Variable	Frequency	Percentage
Gender:		
Male	76	16
Female	408	84
BMI:		
<24.9	174	36
25-29.9	125	26
≥30	185	38
Nationality:		
Saudi	475	98
Non-Saudi	09	02
City of Residence:		
Hail	440	93
Other than Hail	35	07
Educational Level:		
School	02	0.4
Middle School	12	2.6
High School	89	18
Bachelors	367	76
Masters or Doctorate	14	03

**Table 2:** Personal information of respondents (n = 484)

Variable	Frequency	Percentage
Skin Type:		
Oily	149	31
Dry	79	16
Combination	256	53
Frequency of using facial cleanser:		
Never	113	23
1-2 times/week	124	25
3-4 times/week	144	30
1-2 times/day	105	22
Sun-Block Cream:		
Regular user	140	29
Do not use	344	71
Chronic Disease:		
Yes, have	43	09
Do not have	441	91
Smoking:		
Yes	26	05
No	453	94
Ex-smoker	05	01
Physical Exercise (hours/week):		
Never	270	56
1-2 times	135	28
3-4 times	50	10
Every day	29	06
Sleep (hours/day):		
Less than 5	32	07
5-7	61	13
7-9	240	50
More than 9	151	13
Menstrual Cycle (n = 408 females):		
Regular	129	32
Irregular	279	68
NA (male-respondents)	76	

As shown in Table 3, 65% (316/484) of our respondents were suffering from mild to moderate acne. Out of them, majority (81%) had acne on whole face. Majority (57%) was applying topical ointments. Seventy-four percent of respondents had mainly mothers and sisters affected by acne. Table 4 reveals that majority (72%) of the respondents had the knowledge that stress, fatigue, tension are the main causes of acne followed by hormonal abnormality, application of oily creams and cosmetics. Sixty percent answered that acne is a hereditary skin problem, though excessive sweating, obesity, closed-pores and improper cleaning of skin were also marked as causative factors by majority (Table 5). Majority of respondents (70%) had the opinion that fried foods and chips aggravate acne (Table 5).

**Table 3:** Information regarding respondents’ acne

Variable	Frequency	Percentage
Having Acne (n = 484):		
Yes	316	65
No	168	35
Site of Acne (n = 316):		
Whole face	277	81
Whole face with chest	21	13
Whole face with chest and back	09	03
Face with chest, back and upper arm	09	03
Severity of your acne (n = 316):		
Mild	121	38
Moderate	135	43
Severe	60	19
Visited any doctor for acne (n = 316):		
Yes	200	63
No	136	37
Treatment for acne (n = 316):		
Oral medication	50	16
Topical medication	181	57
Face wash	50	16
None	35	11
Any family member affected with acne (n = 316):		
Yes	234	74
No	82	26
If yes, what is your relation? (n = 234)		
Father	10	04
Mother	56	24
Sibling	155	66
Own children	13	06

**Table 4:** Relationship of acne with certain variables (n = 484)

Variable	Frequency	Percentage
Stress & Fatigue:		
Yes	350	72
No	110	23
I do not know	24	05
Hormonal Abnormality:		
Yes	301	62
No	18	04
I do not know	165	34
Scalp Oil or Bleaching Cream:		
Yes	288	60
No	186	38
I do not know	10	02
Cosmetics:		
Yes	263	55
No	201	42
I do not know	20	03

**Table 5:** Aggravating factors for acne (n = 484) (more than one options were selected by respondents)

Variable	Frequency	Percentage
Conditions that aggravate acne:		
Not cleaning the skin properly	145	30
Constant exposure to sunlight and humidity	121	25
Exercise/excessive sweating	08	40
Hijab border sites	01	05
Lack of sleep	06	28
Removal of hair by wax or shave	92	19
Closed pores	145	30
Obesity	08	40
Hereditary	290	60
Foods that aggravate acne:		
Fatty food and butter	145	30
Chocolate	218	45
Dairy products	242	50
Soft drinks	145	30
Fried food or chips	339	70

Dietary habit of those who had acne is shown in Table 6. Majority of them used to drink whole milk 3-4 times/week. Similarly, dairy products were also consumed by the same frequency. Majority of them took twice a week fruit, fruit juices and vegetables. Chocolates, fast foods, oily foods and sea-foods were also taken by the same frequency by the respondents. Nuts were taken quite often by 37% of respondents.

**Table 6:** Dietary history of respondents who had acne (n = 316)

Variable	Frequency	Percentage
Milk: Never	09	03
1-2 times/week	126	40
3-4 times/week	101	32
1-2 times/day	74	25
Type of milk: Whole	245	78
Skimmed	71	22
Dairy Product: Never	06	02
1-2 times/week	91	28
3-4 times/week	119	38
Every day	100	32
Fruits: Never	06	02
1-2 times/week	290	92
3-4 times/week	10	03
Every day	10	03
Vegetables: Never	11	04
1-2 times/week	182	03
3-4 times/week	113	57
Every day	10	36
Sea Food: Never	46	15
1-2 times/week	180	57
3-4 times/week	80	25
Every day	10	03
Chocolate: Never	14	04
1-2 times/week	119	37
3-4 times/week	83	26
Every day	100	33
Nuts: Never	20	06
1-2 times/week	128	41
3-4 times/week	118	37
Every day	50	16
Oily Food: Never	28	09
1-2 times/week	142	45
3-4 times/week	120	38
Every day	26	08

Table 7 reveals association of occurrence of acne with certain variables. Here age between 21-25 years and oily skin have a highly significant association (p = 0.000) with development of acne. Other variables that showed significant association with acne are being obese, smoker, stressful, irregular menstrual cycles and excessive intake of nuts.

**Table 7:** Association of occurrence of acne with certain variables (chi-square test considering level of significance ≤ 0.05)

Comparing Variable	Occurrence of Acne
	p-value
Age between 21-25 years	0.000
BMI (≥30)	0.005
Skin Type (Oily)	0.000
Smoking	0.003
Being Stressful	0.040
Intake of Dairy Products	0.913
Intake of Chocolates	0.099
Doing Physical Exercise	0.917
Irregular Menstruation	0.027
Intake of Fast Food	0.171
Excessive Intake of Nuts	0.025

## Discussion

Acne vulgaris is a common chronic skin disease involving blockage of and/or inflammation of pilosebaceous units. This study was conducted to determine the epidemiology of acne vulgaris and its association with lifestyle among adolescents and young adults. In the current study, the age group of the participants ranged from 10 to 35 years. Before discussion of our results, limitations of the study include the cross-sectional design, which makes interpretation of causality difficult, and the use of self-reported data on BMI and acne, which can allow for measurement errors.

The prevalence of acne in this study was found to be 65% among adolescents and young adults in Hail, Saudi Arabia. Highly significant association was found (p-value = 0.000) between acne occurrence and age between 21-25 years. Similar results were reported in a study conducted in Jeddah, Saudi Arabia, which found that overall prevalence of acne among males and females was 64.5% [15]. Also, we are in accordance with another study that was conducted in Central Saudi Arabia which showed that 56.2% of the university students had acne [16]. In addition, we are in accordance with similar study that was conducted in the Northern region of Saudi Arabia, which reported that the prevalence of acne vulgaris was 53.5% among adolescent male students [6]. There is uniformity in the rates reported in recent studies regardless of the variation of the assessment tools used. Our results are much higher than the results reported in an Australian study which reported that overall prevalence of acne among male and female participants was 36.1% [17]. In addition, our results are slightly higher than the results of a study in Makkah, Saudi Arabia, which found the prevalence of acne among teenage females was 45.7% [18]. However, our findings are much lower than the findings of a study in Tehran where the overall prevalence of acne was 93.2%, 94.4% for boys and 92.0% for girls [13]. The very high rate of acne vulgaris in Iranian adolescents could be explained by genetic predisposition, their skin nature has a tendency to develop acne, certain nutrition habits in the society that increase formation of acne. Also, the study population had pupils aged 16 +/- 0.9 years, which is the highest affected group due to increase sebaceous glands activity in this age. However, the high prevalence in our study may be due to the fact that our study was conducted on adolescents and young adults and these age groups are more commonly affected by acne.

Moreover, the difference in prevalence rates between these studies might be affected by different diagnostic criteria. In this study, the prevalence of respondents who had BMI >30 was 38% and there was significant association (p-value = 0.005) between occurrence of acne and high BMI. Few and controversial data are available concerning the relationship between BMI and acne. Similar results in a study conducted in Taiwan among schoolchildren (aged 6-11 years), reported the mean BMI in students with acne was much higher than that of unaffected students with no specific gender preference [19]. In addition, in a study conducted in Saudi Arabia among women with polycystic ovary syndrome, acne was associated with overweight and obesity [20]. Also, we are in accordance with a study conducted in Eastern Saudi Arabia among obese female schoolchildren which reported that the prevalence of acne in the obese was 30.9% [21].

It was hypothesized that obesity can cause peripheral hyperandrogenism, which may lead to increased sebum production and the development of severe acne. On the contrary, a study conducted in the United Kingdom within the Glasgow Alumni Cohort found no any association between acne and BMI [22]. Conversely, a study conducted in Taiwan investigating the impact of obesity on cutaneous manifestations of clinical hyperandrogenism, found that obese women had less acne than nonobese subjects, although BMI had a significant positive correlation with serum total testosterone [23]. Our data show a main protective effect on the appearance of acne from lower BMI values. The prevalence of skin type in this study was oily (31%), dry (16%) and combination (53%). Our study showed highly significant association (p-value = 0.000) between oily skin and acne.

Another study conducted among adolescents and young females in Arar, Saudi Arabia showed the prevalence of greasy skin among acne patients was 72.7% and dry skin was 27.3% [6]. In addition, a study was done among acne patients in a suburban population that found the prevalence of oily skin was 61.4% and there is a relation between severity of acne and oiliness [24]. Kulthanan et al. reported two-thirds of acne patients to have oily skin [25]. The association between acne and oily skin may be explained by increased sebum secretion and it is a major concurrent event associated with the development of acne.

There are few studies about relationship between acne and smoking. Smoking can lead to adverse effects on the skin due to alterations in the skin microcirculation, keratinocytes, and collagen and elastin synthesis. Smoking could cause alteration in sebum composition through decrease antioxidants. We found significant association (p-value = 0.003) between smoking and appearance of acne. Similar results in a study conducted in Italy showed a correlation between smoking and acne [26]. On the contrary, a study done in Italy among adolescents and young adults showed no association between smoking and acne [10]. There are not enough studies that investigated the relationship between acne and physical activity. In this study, there is no significant association (p-value = 0.913) between doing physical exercise and acne. In agreement with a study conducted in Tehran, it reported no association between physical activity and acne [13]. Hormonal factors play a role in acne development, but few studies have been done to analyze the role of menstrual history on the risk of acne. We found significant association (p-value = 0.027) between menstrual disturbance and acne. We are in accordance with a study conducted in Makkah that showed association between irregular menstruation and acne as 80% of acne patients had menstrual disturbance [18]. Conversely, a study conducted in Italy reported no relationship between menstrual pattern and acne [10]. Also, a large scale twins study conducted in the United Kingdom showed no relationship between acne and reproductive or hormonal potential risk factors [27]. As when we asked about the severity of the participants’ acne 43% reported it as moderate severity, and 38.9% as mild which correlate with Alfalogy study as they display 38% were moderate in females and 30% were mild [18]. Similarly, in another study aimed to evaluate the effect of practice toward lifestyle in adolescence it showed 70 students out of 148 had variable degrees of severity and the overall was 47.3% [28]. Participants’ knowledge of the relation between acne and stress is really impressing (72%) with significant p-value = 0.040. While 58% thought that stress would make acne worse in Jeddah study but with no significant value [15]. Another study emphasizes that stress is a risk factor for acne with p-value of <0.001 and almost 82% of participants [18]. Dairy products are thought to aggravate acne, however, the participants’ response was 30% with no significance, 38% took it 3 or 4 times/week and the majority took the whole milk (78%). While a study in Italy showed that milk products increase the risk of acne especially skim milk more than the whole milk [10]. Also, Al Hussein et al.’s study showed that milk has no effect on acne with p-value of 0.938 [28].

## Conclusions

This study was focused on Hail population in Saudi Arabia to reveal the prevalence of acne vulgaris, and it found that more than half of participants had acne in the age between 21-25 which is an increase in the incidence of the disease and it is worrisome. More workup is needed now for assessing the cause, the level of awareness, and addressing more risk factors to draw attention on the necessity for the development of effective intervention program with multidisciplinary teams (dermatologist, health educator, psychologist, social worker, and physical trainer) working in harmony to control, reduce, and properly manage the burden of the disease.

This program should include a comprehensive school-based acne education program, and a university-based acne education program should be considered for acne vulgaris students and their parents to improve the awareness about young adulthood acne disease. Meanwhile, determining most causative agents for adults and young adults will help dermatologist to educate the patients regarding environmental modification.

## References

[1] Vos T, Flaxman AD, Naghavi M (2012). Years lived with disability (YLDs) for 1160 sequelae of 289 diseases and injuries 1990-2010: a systematic analysis for the Global Burden of Disease Study 2010. The Lancet.

[2] ‏Hay RJ, Johns NE, Williams HC (2014). The global burden of skin disease in 2010: an analysis of the prevalence and impact of skin conditions. J Invest Dermatol.

[3] Degitz K, Placzek M, Borelli C, Plewig G (2007). Pathophysiology of acne (Article in German). J Dtsch Dermatol Ges.

[4] Al Shammrie F, Al Shammrie A (2017). Pattern of skin disease in Hail region of Saudi Arabia. J Dermatol Dermatol Surg.

[5] Parthasaradhi A, Al Gufai AF (1998). The pattern of skin diseases in Hail region, Saudi Arabia. Ann Saudi Med.

[6] Abo El-Fetoh NM, Alenezi NG, Alshamari NG, Alenezi OG (2016). Epidemiology of acne vulgaris in adolescent male students in Arar, Kingdom of Saudi Arabia. J Egyptian Public Health Assoc.

[7] Alanazi MS, Hammad SM, Mohamed AE (2018). Prevalence and psychological impact of Acne vulgaris among female secondary school students in Arar city, Saudi Arabia, in 2018. Electron Physician.

[8] Mahmood SN, Bowe WP (2014). Diet and acne update: carbohydrates emerge as the main culprit. J Drugs Dermatol.

[9] Ballanger F, Baudry P, N’Guyen J, Khammari A, Dréno B (2006). Heredity: a prognostic factor for acne. Dermatology.

[10] Di Landro A, Cazzaniga S, Parazzini F (2012). Family history, body mass index, selected dietary factors, menstrual history, and risk of moderate to severe acne in adolescents and young adults. J Am Acad Dermatol.

[11] Berra B, Rizzo AM (2009). Glycemic index, glycemic load, wellness and beauty: the state of the art. Clin Dermatol.

[12] Fiedler F, Stangl GI, Fiedler E, Taube K-M (2017). Acne and nutrition: a systematic review. Acta Derm Venereol.

[13] Ghodsi SZ, Orawa H, Zouboulis CC (2009). Prevalence, severity, and severity risk factors of acne in high school pupils: a community-based study. J Invest Dermatol.

[14] Campbell GG (1931). The relation of sugar intolerance to certain diseases of the skin. Br J Dermatol.

[15] Al Mashat S, Al Sharif N, Zimmo S (2013). Acne awareness and perception among population in Jeddah, Saudi Arabia. J Saudi Soc Dermatol Dermatol Surg.

[16] Al Robaee AA (2005). Prevalence, knowledge, beliefs and psychosocial impact of acne in University students in Central Saudi Arabia. Saudi Med J.

[17] Stathakis V, Kilkenny M, Marks R (1997). Descriptive epidemiology of acne vulgaris in the community. Australas J Dermatol.

[18] Alfalogy EH (2018). Epidemiology of acne vulgaris: prevalence, severity and its impact among school teenagers in Makkah, Saudi Arabia. Egyptian Family Med J.

[19] Tsai M-C, Chen W, Cheng Y-W, Wang C-Y, Chen G-Y, Hsu T-J (2006). Higher body mass index is a significant risk factor for acne formation in schoolchildren. Eur J Dermatol.

[20] Tamimi W, Siddiqui IA, Tamim H, AlEisa N, Adham M (2009). Effect of body mass index on clinical manifestations in patients with polycystic ovary syndrome. Int J Gynecol Obstet.

[21] Al-Saeed WY, Al-Dawood KM, Bukhari IA, Bahnassy A (2006). Dermatoses in obese female schoolchildren in the Al-Khobar area, Eastern Saudi Arabia. J Family Community Med.

[22] Galobardes B, Smith GD, Jeffreys M, Kinra S, McCarron P (2005). Acne in adolescence and cause-specific mortality: lower coronary heart disease but higher prostate cancer mortality: the Glasgow Alumni Cohort Study. Am J Epidemiol.

[23] Yang J-H, Weng S-L, Lee C-Y, Chou S-Y, Hsu C-S, Hsu M-I (2010). A comparative study of cutaneous manifestations of hyperandrogenism in obese and non-obese Taiwanese women. Arch Gynecol Obstet.

[24] Hazarika N, Rajaprabha RK (2016). Assessment of life quality index among patients with acne vulgaris in a suburban population. Indian J Dermatol.

[25] Kulthanan K, Jiamton S, Kittisarapong R (2017). Dermatology life quality index in Thai patients with acne. Siriraj Med J.

[26] Capitanio B, Sinagra JL, Ottaviani M, Bordignon V, Amantea A, Picardo M (2009). Acne and smoking. Dermatoendocrinol.

[27] Bataille V, Snieder H, MacGregor A, Sasieni P, Spector T (2002). The influence of genetics and environmental factors in the pathogenesis of acne: a twin study of acne in women. J Invest Dermatol.

[28] Al Hussein SM, Al Hussein H, Vari CE, Todoran N, Al Hussein H, Ciurba A, Dogaru MT (2016). Diet, smoking and family history as potential risk factors in acne vulgaris - a community-based study. Acta Medica Marisiensis.

